# Human rhinovirus infection of human bronchial epithelial cells results in migration of human airway smooth muscle cells

**DOI:** 10.1186/1710-1492-10-S1-A69

**Published:** 2014-03-03

**Authors:** Sami Shariff, Sergei Nikitenko, Abid Qureshi, Jason Arnason, Chris Shelfoon, Suzanne Traves, David Proud, Richard Leigh

**Affiliations:** 1 Snyder Institute for Chronic Diseases, University of Calgary, Calgary, Alberta, T2N 4N1, Canada

## Background

Young children who experience human rhinovirus (HRV)-associated wheezing illnesses are more likely to develop subsequent asthma [1]. This has led to the hypothesis that HRV infection may be involved in the pathogenesis of airway remodeling in asthma [2]. Increased airway smooth muscle (ASM) mass, in which ASM cells are in close proximity to the subepithelial region, is a characteristic feature of airway remodeling [3]. We have shown that HRV infection of human bronchial epithelial (HBE) cells, both *in vitro* and *in vivo*, results in the upregulation of airway remodeling mediators [4]. We now sought to determine whether HRV infection of HBE cells is associated with airway smooth muscle (ASM) chemotaxis.

## Methods

Primary HBE cells pre-treated with medium containing 1% insulin, transferrin, and selenium (ITS) for 24 hours were exposed to medium (control) or purified HRV-16 (MOI: 0.3-1) for 24 hours. HBE cell supernatants were then studied as chemoattractants for ASM chemotaxis. ASM migration was examined using both a 48-well modified Boyden chamber and a 16-well xCELLigence^®^ apparatus (Roche, Laval, Canada). In the latter case, migration to HBE supernatants was measured via electrical impedance, as per manufacturer’s instructions, and compared to medium control. Chemotactic gradient was abolished by addition of chemoattractants to top and bottom wells of the apparatuses. Additionally, the effects of filtered supernatants and pre-treatment with Pertussis toxin (PTX) on ASM chemotaxis were studied using the xCELLigence^®^ apparatus.

## Results

Supernatants from HRV-16 infected HBE cells resulted in significantly greater ASM cell directional migration, compared to supernatants from HBE cells exposed to medium (negative control) in both the Boyden chamber (n=3; p<0.01) and the xCELLigence system (n=3; p<0.001); relationship was concentration-dependent (n=3; p<0.05). Time course experiments demonstrated that rate of migration was maximal at 4 hours. Migration was significantly attenuated when the chemotactic gradient was abolished, indicating that directional cell migration is due to chemotaxis and not chemokinesis (n=3; p<0.001). ASM migration significantly increased even after filtration of HRV and proteins greater than 100,000 MW (n=3; p<0.05). However, pretreatment with PTX abrogated HRV-induced ASM chemotaxis (n=3, p<0.05). Pretreatment with dexamethasone, formoterol, or combination, abolished ASM chemotaxis (n=4, p<0.05).

## Conclusions

These data provide the first demonstration that HRV infection of airway epithelial cells produces soluble factor(s) that cause directional migration of ASM cells. Moreover, migration appears to be dependent on a PTX sensitive pathway. Taken together, they provide further evidence for a role of HRV infection in the pathogenesis of airway remodeling in asthma.
